# Mechanisms of sensorineural cell damage, death and survival in the cochlea

**DOI:** 10.3389/fnagi.2015.00058

**Published:** 2015-04-21

**Authors:** Ann C. Y. Wong, Allen F. Ryan

**Affiliations:** ^1^Department of Surgery/Division of Otolaryngology, University of California, San Diego School of MedicineLa Jolla, CA, USA; ^2^Department of Physiology and Translational Neuroscience Facility, School of Medical Sciences, University of New South WalesSydney, NSW, Australia; ^3^Veterans Administration Medical CenterLa Jolla, CA, USA; ^4^Department of Neurosciences, University of California, San Diego School of MedicineLa Jolla, CA, USA

**Keywords:** presbycusis, age-related hearing loss (ARHL), hair cells (HCs), spiral ganglion neurons (SGN), reactive oxygen species (ROS), c-Jun terminal kinase (JNK), inflammation, cell survival signaling

## Abstract

The majority of acquired hearing loss, including presbycusis, is caused by irreversible damage to the sensorineural tissues of the cochlea. This article reviews the intracellular mechanisms that contribute to sensorineural damage in the cochlea, as well as the survival signaling pathways that can provide endogenous protection and tissue rescue. These data have primarily been generated in hearing loss not directly related to age. However, there is evidence that similar mechanisms operate in presbycusis. Moreover, accumulation of damage from other causes can contribute to age-related hearing loss (ARHL). Potential therapeutic interventions to balance opposing but interconnected cell damage and survival pathways, such as antioxidants, anti-apoptotics, and pro-inflammatory cytokine inhibitors, are also discussed.

## Introduction

### Acquired Sensorineural Hearing Loss (SNHL)

Sensorineural hearing loss (SNHL) is a common sensory deficit, the WHO estimating in 2012 that globally over 360 million people have disabling hearing loss (Duthey, 2013). SNHL is often defined as the loss of hearing sensitivity due to peripheral tissue damage and/or cell death in the hearing organ, the cochlea. However, it is increasingly recognized that the central auditory structures of the brain can also play an independent role in SNHL. The etiology of SNHL is multifactorial, with overexposure to sound, certain drugs, infection or immune-induced inflammation being common causes. Perhaps the most common form of hearing loss is presbycusis, age-related cochlear or central auditory tissue damage or degeneration, often aggravated by other factors including a history of noise exposure, diabetes or high blood pressure. These factors lead to damage and death of the sensory receptor cells in the cochlea and the neurons that relay auditory information from the inner ear to the central auditory circuitry, and then process information as it ascends the central auditory pathway. Damage restricted to the central auditory pathway also plays a role in age-associated hearing loss (e.g., Ouda et al., 2015).

The sensorineural tissues of the cochlea have very limited repair capacity. The mature cochlear receptor cells, the inner and outer hair cells (HCs), as well as the cochlear neurons do not regenerate, making any cellular loss permanent. An overarching aim in preclinical auditory research is to identify the myriad of cellular molecular mechanisms impacting the damage and survival of cochlear sensorineural tissue, so as to translate into avenues for therapeutic interventions. Here, we review the mechanisms of cochlear HC and neuronal loss and survival, with focus on the role of intracellular pathways and immunity/inflammation in hearing loss. This includes presbycusis, since there is evidence that sensorineural cell damage occurs via similar mechanisms in most forms of SNHL. Moreover, at least some presbycusis is thought to represent the accumulation of hearing loss due to various etiologies over a lifetime.

### Cochlear Structure and Function

A newborn human cochlea has ~3500 inner HCs and a total of ~12000 outer HCs, while the mouse, an important experimental animal in hearing research has ~3300 HCs in one cochlea (Ehret and Frankenreiter, 1977). Cochlear HCs are interdigitated with supporting cells to form an epithelial layer on top of the basilar membrane, these receptor cells are bound by two different cellular fluids: apical stereocilia bath in high K^+^ endolymph and its basal synaptic pole in perilymph with ionic concentration similar to extracellular fluid.

**Figure 1:**
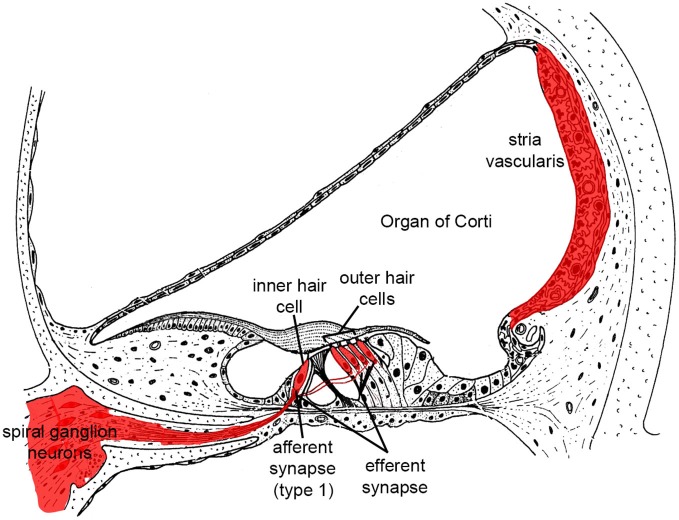
**Illustration highlighting cochlear tissues that are prone to irreversible damage and cell death in SNHL in red**. Damage to any of these tissues reduces hearing.

Sound energy produces fluidic pressure differences across the basilar membrane upon which the sensory cells sit, leading to membrane motion and deflection of bundles of stereocilia on their apical surfaces. This in turn activates mechanoelectrical transduction (MET) channels leading to K^+^ influx and HC receptor potentials. Depolarization of outer HCs produces electromotility and amplification of basilar membrane motion, which forms the basis for cochlear sensitivity and frequency resolution. Depolarized inner HCs release glutamate from ribbon synapses at their basal poles. Innervating the inner HCs are dendrites of the afferent neurons of the cochlear nerve, known as spiral ganglion neurons (SGN). These bipolar neurons provide afferent auditory neurotransmission to the central auditory system. There are ~35,000 afferent SGNs in one human cochlea and ~7000 in a C57 mouse cochlea (Schettino and Lauer, 2013). Cochlear HCs and SGNs are the foundation of hearing function, and as noted above they do not regenerate after death. It is therefore important to curb the cellular mechanisms that contribute to HC and SGN death (Figure 1).

### SNHL and HC Loss

The most common histological sign of SNHL in humans is damage to or the loss of cochlear sensory cells (Schuknecht et al., 1973). This phenomenon has been well studied in animals. For example, very intense noise can cause direct mechanical disruption of the HC stereocilia bundles (Liberman and Beil, 1979; Slepecky, 1986; Patuzzi et al., 1989), reducing or eliminating their function. Even more intense stimulation can disrupt the cellular organization of the sensory epithelium, producing profound damage directly or indirectly via potassium exposure of the HC basal poles. However, most damaging levels of noise are below the threshold of mechanical damage and produce the loss of HCs via biochemical pathways within the cells themselves. Such pathways are also involved in HC loss due to non-traumatic causes. These include ototoxic drugs, such as the aminoglycoside antibiotics (Schacht, 1986; Hiel et al., 1992; Richardson et al., 1997; Alharazneh et al., 2011), loop diuretics or some anticancer medications. Most critical for this review, they are important mediators of age-related hearing loss (ARHL).

### Cochlear Neural Contributions to SNHL

Afferent SGNs depend at least partially for their survival upon the support of neurotrophic factors including neurotrophin-3 (NT-3), brain-derived neurotrophic factor (BDNF), and glial cell line-derived neurotrophic factor (GDNF), released from the HCs and supporting cells of the cochlear sensory epithelium (Schimmang et al., 2003; Sun and Salvi, 2009; Barclay et al., 2011). When HCs die, the afferent dendrites of SGNs retract, and secondary loss of SGN somata is observed (Liberman and Kiang, 1978; Spoendlin, 1984; Sugawara et al., 2005; Kujawa and Liberman, 2009; Moser et al., 2013). Recent evidence from ototoxic and age-related SNHL suggests that SGN damage or loss can be independent from HC loss. SGN afferent terminals and somata have been found to be primary injury sites after carboplatin treatment (Wang et al., 2003a). Similarly, with loud sound injury, SGN afferent terminals juxtaposed to the inner HCs swell immediately after exposure and prior to observable HC damage likely due to glutamate toxicity (Robertson, 1983; Puel et al., 1998). Kujawa and Liberman (2009) found that noise exposure at levels too low to produce permanent threshold shift can nevertheless cause the loss of a substantial number of the afferent synapses between inner HCs and SGNs. Moreover, they also found that such noise exposure potentiated age-related SNHL, also by disrupting cochlear ribbon synapses immediately post noise exposure, leading to selective SGN axonal fiber loss without destroying cochlear sensory cells (Kujawa and Liberman, 2009; Furman et al., 2013). In aged mice never exposed to loud noise, afferent SGN-inner HC innervation reduction was observed before SGN loss, supporting a role for SGNs in addition to HCs as a primary site of age-related SNHL (Sergeyenko et al., 2013). The mechanisms responsible for inner HC synapse loss have not been determined with certainty, but glutamate toxicity is a strong candidate.

On the central projection of SGN axons, electron microscopy analysis showed with repeated exposure to loud noise (100 dB SPL, 3 h per day over 3 days) marked elongation of the nodes of Ranvier and a moderate retraction of the paranodes was observed together with a reduced number of lamellar wraps and decreased myelin thickness (Tagoe et al., 2014). These morphological changes parallel physiological measurements of reduced auditory nerve conduction velocity and increased auditory thresholds.

### Presbycusis

ARHL or persbycusis is most frequently associated with damage to HCs and SGNs. It has been suggested that a substantial contribution to presbycusis is accumulated, low-level damage due to noise and other insults (Gates and Mills, 2005). However, even in the absence of such stimuli, as when animals spend their lives in silence, ARHL is still observed (Sergeyenko et al., 2013; Yan et al., 2013). Damage to other tissues, in particular the stria vascularis, is also associated with aging (Schuknecht, 1964; Fetoni et al., 2011). Given the highly active and metabolic nature of many cochlear tissues, and their delicacy, it is perhaps not surprising that age results in damage independent of extrinsic causes. The inbred mouse model of C57BL/6J background has shown high-frequency hearing loss and cochlea basal turn cytostructural degeneration from 6-months of age, in part caused by the presence of the ARHL susceptibility allele (*Ahl*), which encodes for caderhin 23 expressed in the stereocilia of HCs (Zheng et al., 1999). Fransen et al. (2015) performed a genome-wide association study to identify the genes responsible for ARHL, and found no indication for the presence of any major genes. However, independent post-association pathway analysis suggested the involvement of the arachidonic acid secretion pathway, the JAK/STAT and TGFβ signaling pathways though the authors concluded the findings remain exploratory.

The central auditory system is also subject to changes related to age. Many of the alterations that have been documented in the aging central auditory system are related to the expression of potassium channels (Frisina, 2009) and neurotransmitters (Ling et al., 2005; Wang et al., 2009). For example, Zettel et al. (2007) found declines in Kv3.1 in the auditory midbrain structures of aging mice, which could contribute to reduced discharge and synchrony of auditory evoked neural activity. Similarly, Caspary et al. (2008) and Ouda and Syka (2012) noted reduced glycine and GABA expression in auditory midbrain structures with age, accompanied by altered expression of their receptors. This would alter the excitatory/inhibitory balance of auditory neurons and affect signal processing. Such changes are likely to alter the transmission of information in the ascending auditory pathway, and presumably contribute to altered perception of sounds that are independent of peripheral changes in thresholds. They can also influence the cochlea. Jacobson et al. (2003) observed dramatic declines in contralateral suppression known to be mediated by the olivocochlear efferents, in aged mice.

## Intracellular Mechanisms of HC Loss

### Free Radical Accumulation and Calcium Homeostasis

Generation of reactive oxygen species (ROS) and reactive nitrogen species (RNS) in the cochlea are triggered by exposure of loud sound and ototoxic drugs often followed by caspase-mediated cell death by apoptosis (Shi and Nuttall, 2003; Henderson et al., 2006; Hu et al., 2006). ROS have been detected in cochlear tissue immediately after noise exposure (Yamane et al., 1995) and seen to persist for 7–10 days after, spreading from the basal end of the organ of Corti to the apical turn; the RNS product peroxynitrite (ONOO−), generated by the combination of nitric oxide (NO) and superoxide has also been found (Yamashita et al., 2004a). This prolonged oxidative stress can induce the delay and continued cochlear injury. Apoptosis-inducing factor (AIF) and the apoptotic nuclease EndoG are also released by mitochondria into the cytosol of cochlear HCs following noise exposure (Yamashita et al., 2004b). Translocation of these pro-apoptotic factors into the nucleus triggers apoptosis. Activation of the c-Jun *N*-terminal kinase/mitogen-activated protein kinase (JNK/MAPK) signaling pathway is also implicated in outer HC apoptosis in response to oxidative stress (Wang et al., 2007).

Free radicals (ROS and RNS) can cause damage by reacting with DNA, proteins, cytosolic molecules, cell surface receptors, and breaking down membrane lipids. ROS produced by the mitochondria induce lipid peroxidation in the cochlea through the formation of malondialdehyde and 4-hydroxynonenal byproducts (Yamashita et al., 2004a). This overloads the cochlear antioxidant enzyme system, including superoxide dismutase (SOD), catalase (CAT), glutathione peroxidase and glutathione reductase, and depletes glutathione, the endogenous antioxidant. In addition to apoptosis, ROS generation also leads to inflammation, and production of the pro-inflammatory cytokines interleukin-6 (IL-6; Wakabayashi et al., 2010) and tumor necrosis factor α (TNFα; Keithley et al., 2008). The presence of vasoactive lipid peroxidation products such as isoprostanes potentially also leads to the reduced cochlear blood flow associated with excessive noise (Thorne et al., 1987; Seidman et al., 1999; Ohinata et al., 2000; Jaumann et al., 2012). Noise-induced ischemia and subsequent re-perfusion further potentiate the generation of ROS. Expression of nicotinamide adenine dinucleotide phosphate (NADPH) oxidases, a key component in the cellular antioxidation system, is altered in the HCs and supporting cells of noise-exposed cochleae (Vlajkovic et al., 2013). Maulucci and colleagues have shown further that acoustic overstimulation reduced NADPH oxidation, decreasing plasma membrane fluidity of outer HCs as a consequence of excess peroxidation induced by an altered metabolic state (Maulucci et al., 2014). Interestingly, Fetoni and colleagues have recently identified endogenous coenzymes Q_9_ and Q_10_ to be reduced in the cochlea after exposure to 60 min of 100 dB SPL sound for 10 consecutive days (Fetoni et al., 2013). Systematic administration of exogenous CoQ_10,_ beginning 3 days prior to the noise insult, has been shown to improve hearing threshold, reducing HC bundle disorientation and SGN loss in the mid-cochlear turn, likely due to lowered noise-induced oxidative stress on the sensorineural tissues. Accumulation of highly-reactive oxygen species (hROS) in HCs is also found in cultured organ of Corti explants exposed to gentamicin (Choung et al., 2009).

Mitochondria are a potent source of ROS and loss of mitochondrial integrity can lead to increased ROS production and release of ROS into the cytoplasm (Batandier et al., 2004). Recent work by Esterberg and colleagues suggests that aminoglycoside antibiotics may induce cytoplasmic ROS in HCs by disrupting calcium homeostasis between the endoplasmic reticulum (ER) and mitochondria (Esterberg et al., 2013, 2014). Their data suggest that these drugs enhance calcium flow into mitochondria, leading to increases in mitochondrial membrane permeability, release of calcium as well as increased ROS production and release into the cytoplasm, with subsequent HC damage and death.

Excessive noise also leads to an increase in free Ca^2+^ in cochlear HCs immediately post-noise (Fridberger et al., 1998). This increase can be caused by Ca^2+^ entry through ion channels, such as L-type Ca^2+^ channels an P2X_2_ ATP receptor subunit, and lead to further release of Ca^2+^ from intracellular stores such as the ER and mitochondria (Orrenius et al., 2003). Elevated Ca^2+^ levels in the cochlea may link to ROS production as well as triggering apoptotic and necrotic cell death pathways independent of ROS formation (Orrenius et al., 2003). In knock-out mice lacking expression of the canonical transient receptor potential channel subtype 3 (TRPC3), a non-selective cation-permeable receptor expressed in sensorineural cochlear tissue (Raybould et al., 2007; Phan et al., 2010; Tadros et al., 2010), cochlear HCs displayed approximately 40% reduction in Ca^2+^ re-entry following intracellular calcium depletion. The TRPC knockout mice have hyperacusis at frequencies tonotopically encoded by mid-apical basilar membrane, a region highly reliant on outer HC cochlear amplification (Wong et al., 2013b). The consequence of disrupted calcium homeostasis on noise susceptibility is also demonstrated in plasma membrane Ca^2+^-ATPase isoform 2 (*Pmca2* or *Atp2b2*) mutant mice. The *C*-terminally truncated PMCA2a is the only isoform detected in the stereocilia of HCs (Furuta et al., 1998; Dumont et al., 2001). *Pmca2* null mice are deaf while their heterozygous littermates have significant hearing loss (Kozel et al., 1998). People carrying a homozygous mutation in cadherin 23 (*CDH23*) and a heterozygous, hypofunctional variant in *PMCA2* have exaggerated hearing loss compared to those having *CDH23* mutation alone (Schultz et al., 2005).

Another mode of action of calcium is to modulate the activity of MAPK and other intracellular signaling cascades, including apoptosis (e.g., Agell et al., 2002; Harr and Distelhorst, 2010). There is ample evidence that these cascades play a significant role in damage to both HCs and SGN.

### MAPK Signaling Pathways

There is substantial evidence that sensorineural cell damage and death involve intracellular signaling pathways. MAPKs are important intracellular proteins that, when phosphorylated, regulate diverse cellular processes in response to a variety of extracellular and intracellular stimuli. MAPKs mediate plasma membrane bound receptor signals to activate transcription factors in the nucleus, facilitating gene expression, coordinately regulating cell proliferation, differentiation, motility, and survival (Johnson, 2011). They can also respond to intracellular events such as ROS or intracellular receptors (Torres, 2003). Among this family of signaling proteins, the classical MAPKs include the extracellular signal-regulated kinases 1, 2 and 5 (ERK1, 2 and 5), stress-activated protein kinase/JNK1-3, and p38 (α, β, γ, δ). MAPKs together regulate a large number of substrates, including members of a family of protein Ser/Thr kinases known as MAPK-activated protein kinases (MAPK-APK). Stress-activated protein kinases of the JNK and p38 families are key mediators of stress and inflammation responses evoked by a variety of physical, chemical and biological stress stimuli, while the ERK1/2 cascade is most often induced by growth factors and mediates tissue growth and survival. p38 MAPK activation is a major component deciding cell fate in response to cisplatin, primarily to induce apoptosis (Brozovic and Osmak, 2007).

In animals exposed to intense noise, MAPK phosphorylation in the cochlea is altered, ERK1/2 in the sensory and support cells of the cochlear sensory epithelium are activated by phosphorylation; while JNK and p38 MAPKs showed late activation in the SGN, de novo syntheses of the MAPKs are also observed (Meltser et al., 2010; Jamesdaniel et al., 2011; Maeda et al., 2013; Patel et al., 2013). Radiation therapy for the treatment of head and neck cancers produces severe ototoxicity due to the increased production of ROS. In a cochlea derived cell line model, pharmacological inhibition of p38 prior to radiation exposure has prevented radiation ototoxicity (Shin et al., 2014). Inhibitors of JNK have demonstrated protection against noise-induced and aminoglycoside-induced HC loss (Pirvola et al., 2000; Wang et al., 2003b, 2007). Inhibition of upstream activators of the JNK pathway, including kRas, Rac/cdc42 and mixed lineage kinases also provides protection to HCs (Bodmer et al., 2002a,b; Battaglia et al., 2003).

### Apoptosis

Following the activation of MAPK and ROS stress pathways, cochlear HCs can undergo apoptosis following intense noise exposure, aminoglycoside or cisplatin ototoxicity, or aging (Hu et al., 2000, 2002a,b; Nicotera et al., 2003; Wang et al., 2003b; Yang et al., 2004). Apoptosis can occur through the sequential actions of caspases, initiated by their associated extrinsic and intrinsic pathways (Yakovlev and Faden, 2001). The extrinsic pathway is initiated by extracellular stimuli through the activation of transmembrane death receptors, which cleave caspase-8 and activate the downstream execution pathway mediated by caspase-3. The intrinsic pathway is initiated by a change in mitochondrial membrane permeability, which not only activates caspase-9 and the downstream execution pathway via cytochrome C release, but also releases ROS. The execution pathway results in distinctive structural pathology including cell shrinkage, chromatin condensation, formation of cytoplasmic blebs and apoptotic bodies and finally phagocytosis of the apoptotic bodies by adjacent parenchymal cells, neoplastic cells or macrophages. Alternatively, there are caspase-independent processes that lead to apoptosis, mediated by other factors including receptor-interacting serine/threonine-protein kinase 1 (RIP-1) or AIF (Tait and Green, 2008).

The caspase-mediated cell death pathway has been widely implicated in programmed cell death of HCs (Nicotera et al., 2003; Yang et al., 2004; Bohne et al., 2007; Tadros et al., 2008), with the preponderance of evidence implicating the intrinsic pathway (e.g., Tabuchi et al., 2007; Esterberg et al., 2013, 2014), but evidence also for extrinsic pathway involvement (Bodmer et al., 2002b).

### Infection, Immunity and Inflammation

Invasion of tissue by bacteria or viruses provokes an immediate response based on innate immunity that is independent of prior sensitization, and a delayed response based on acquired, cognate immunity. The processes of inflammation form an important party of both forms of immune defence, generating bioactive mediators that attack or impede infecting organisms, recruiting immune cells to the site and promoting the death of pathogens. However, inflammation is also known to damage or kill host cells via bystander injury. Pro-inflammatory cytokines such as IL-1β and TNFα produce toxicity through multiple pathways including complement activation, production of arachidonic acid metabolites and ROS formation (Neher et al., 2011). TNFα can also activate the extrinsic pathway of apoptosis via TNF “death” receptors common on the surface of cells. Leukocytes drawn to sites of inflammation by cytokines and chemokines may also cause tissue damage. For example, neutrophils are potent sources of ROS, and their release can cause harm to host cells (Wright et al., 2010).

Infection of the inner ear by viruses or bacteria causes varying degrees of inflammation, depending upon the nature of the organism and the host response. Bacterial labyrinthitis, especially when caused by invasive organisms such as *Streptococcus pneumoniae* or *Haemophilus influenzae* type B (Dodge et al., 1984; Wiedermann et al., 1986) is devastating to the delicate tissue of the inner ear, and immunization against these pathogens provides significant protection (Schuchat et al., 1997). On the other hand, there is evidence that the immune response to less damaging pathogens may produce a more severe reaction than the original infection. For example, cytomegalovirus (CMV) infection of the adult cochlea, unlike during the prenatal period, typically causes mild hearing loss and cochlear damage (Juanjuan et al., 2011; Wang et al., 2013). However, Woolf et al. (1985) found that infection of the adult guinea pig cochlea was far more damaging in animals that had previously been immunized systemically with the virus. This indicates that CMV can cause a relatively benign infection in the adult cochlea, while immune-mediated inflammation leads to damage and hearing loss.

Infections outside of the inner ear can also cause cochlear damage. Middle ear infections have been shown to damage especially the basal turn of the cochlea, even when the infection does not enter the labyrinth (Hunter et al., 1996). This is likely due to the relative permeability of the round window membrane, which can allow inflammatory mediators to enter the cochlea. Several studies have found that when such mediators are applied to the round window, they are detected in perilymph and can lead to sensorineural damage and hearing loss (Huang et al., 1990; Kubo et al., 1998).

In addition to the response to infection, tissue damage itself can initiate inflammation, through the release of damage-associated molecules including arachadonic acid metabolites, free extracellular matrix molecules, nucleotides and others (Van Crombruggen et al., 2013). This cochlear damage due to a variety of causes can initiate the inflammatory cascade in the absence of infection. Noise-induced hearing loss has been found to induce inflammation as evidenced by the observation of pro-inflammatory cytokines (Wakabayashi et al., 2010), leukotrienes (Park et al., 2014) and leukocytes (Hirose et al., 2005; Tornabene et al., 2006) within the inner ear. Similarly, ototoxicity can also induce inner ear inflammation (Sato et al., 2008, 2010; Dinh et al., 2013).

Autoimmunity has also been implicated as a mechanism of SNHL. This was originally based upon the observation that some forms of idiopathic hearing loss improve with steroid treatment, and may worsen upon the termination of therapy (Wilson et al., 1980). Identification of cochlear autoimmune antigenic targets has been difficult, although the most recent evidence implicates cochlin, one of the most prevalent proteins in the cochlea (Baruah, 2014). Interestingly, it has been suggested that this may be related to homology between cochlin and proteins present in certain molds that induce innate immune and allergic responses (Pathak et al., 2013).

Of particular interest for this review, an association between systemic inflammation and ARHL has recently been noted in human studies (Verschuur et al., 2012; Nash et al., 2014). A recent review by Franceschi and Campisi (2014) more broadly discusses the relationship between inflammation and aging (“inflammaging”). They cite extensive evidence to support the role of chronic inflammation in the aging process. In particular, low-grade production of pro-inflammatory cytokines including IL-6, IL-1β and TNFα appear to be significant mediators of age-related damage to a wide variety of tissues. Expression of these cytokines is associated with JAK/STAT signaling. As noted above, a genome-wide association study has implicated the JAK/STAT pathway in ARHL (Fransen et al., 2015), which provides further evidence to support a role for chronic inflammation.

## HC Survival Signaling

Many cellular processes that lead to cell damage and death are tightly regulated to reduce unwanted cellular losses, as noted above for apoptosis. Inflammatory damage can be opposed by anti-inflammatory cytokines such as IL-10 and anti-inflammatory cellular phenotypes such as alternatively activated macrophages. Similarly, there are intracellular pro-survival pathways that oppose the death and damage signaling of stress pathways such as ROS, the JNK and p38 cascades.

### ROS-Opposing Mechanisms

Antioxidants react with ROS, reducing them to eliminate or reduce toxicity. Cochlear HCs contain the antioxidant glutathione, which is the substrate by which glutathione transferases detoxify ROS. The levels of glutathione is lower in the basal cochlear turn HCs increasing towards the apex (Sha et al., 2001), which may be one reason why basal turn HCs are more vulnerable to most forms of SNHL. Glutathione peroxidases (GPxs) and SODs are enzymes that alter ROS to less damaging forms. Studies in mice deficient in SOD1 or GPx1 found increased susceptibility to noise, further indicating that natural defense mechanisms against ROS are important to the survival of HCs under stress. Polymorphisms in glutathione transferase genes, which catalyze the response of glutathione with ROS, have been linked to ototoxicity and noise sensitivity in humans (Peters et al., 2000; Lin et al., 2009; Shen et al., 2012).

Bared et al. (2010) compared polymorphisms in genes encoding glutathione S transferase and N-acetyltransferase, another antioxidant enzyme, in normal hearing subjects vs. presbycusis patients. They found significant associations with presbycusis and polymorphisms that reduce enzyme function. Moreover, Angeli et al. (2012) found that presbycusis patients with glutathione transferase gene polymorphisms were associated with preferential high-frequency hearing loss in patients with presbycusis. The evidence strongly suggests that HCs are equipped with multiple mechanisms with which to neutralize ROS. It seems likely that only when these mechanisms have been exhausted do ROS cause significant harm.

ROS accumulation is linked to activation of the JNK signaling pathway (Son et al., 2013). Interestingly, the calcium binding protein calmodulin, which is highly expressed in HCs, preferentially binds to and inhibits kRas in a calcium-dependent manner (Villalonga et al., 2001). This would be expected to reduce the activity of JNK signaling.

### ERK MAPK Pathway

When Battaglia et al. (2003) evaluated the effects of Ras inhibition on ototoxin-induced HC loss, they noted that high levels of the inhibitor FTI-277 were protective, consistent with reducing downstream JNK signaling. However, lower levels of the inhibitor enhanced HC loss. This is related to the differential sensitivity of Ras isoforms to FTI-277. High concentrations inhibit kRas, which leads to JNK activation, while low levels of FTI-277 inhibit hRas, which leads to inhibition of another MAPK, ERK (Sebti and Der, 2003). ERK is associated with cell survival and proliferation in other tissues (Xia et al., 1995; Xue et al., 2000). This finding suggested that ototoxins can activate two distinct and competing cell signaling pathways within HCs. The balance of signaling between these pathways plays a significant role in determining the fate of the cell. ERK signaling is frequently activated by growth factors, which may also explain why several growth factors have been shown to protect HCs from various insults (Endo et al., 2005; Hayashi et al., 2013, 2014). Mutations in the insulin-like growth factor 1 (*igf1*) gene cause syndromic hearing loss in both humans and mice (Cediel et al., 2006), supporting a role of growth factors in protecting hearing. Interestingly, IGF-1 deletion mice display delayed responses to acoustic stimuli that increase along the central auditory pathways, suggesting a central component to growth factor protection.

### PI3K/PKC/AKT Signaling Pathway

Another well-recognized survival signaling pathway is mediated by phosphatidylinositol 3 kinase (PI3K), protein kinase c (PKC) and protein kinase b (also known as AKT). Chung et al. (2006) found that gentamicin exposure increased AKT activation in the organ of Corti, while an inhibitor of AKT reduced HC loss. Brand et al. (2011) found that simvastatin protects HCs against gentamicin toxicity via AKT signaling. These data indicate that ototoxins activate another survival pathway that competes with cell damage signaling.

### Anti-Apoptotic Signaling

As in all cells, the activation of caspases in HCs appears to be tightly regulated to avoid inappropriate cell death. Inhibitor of apoptosis proteins (IAPs) are a family of endogenous caspase inhibitors, with X-linked inhibitor of apoptosis protein (XIAP) responsible for inhibiting caspase-3 and caspase-9 (Obexer and Ausserlechner, 2014). Adeno-associated virus-mediated delivery of XIAP has shown protection against cisplatin ototoxicity (Cooper et al., 2006; Chan et al., 2007). Tabuchi and coworkers have found inhibitors of XIAP to increase caspase-3 activation in gentamicin-treated organ of Corti culture, with increased auditory HC loss (Tabuchi et al., 2007). Their findings suggest that XIAP is normally present in HCs and that it acts to reduce HC apoptosis during gentamicin ototoxicity.

Apoptosis is also regulated by other processes, including the expression of apoptosis-promoting and inhibiting factors of the Bcl2 family. Pro-apoptotic Bcl2 factors such as Bad and Bax promote cell death by increasing mitochondrial membrane permeability, while inhibitory members such as Bcl2 and BclX oppose cell death by stabilizing the mitochondrial membrane. The balance of these factors can alter the outcome of apoptotic signaling. Over-expressing anti-apoptotic Bcl2 factors has been shown to reduce HC death due to ototoxins (Cunningham et al., 2004). Of particular relevance to this review, aged gerbil HCs show reduced expression of Bcl2 and enhanced expression of Bax (Alam et al., 2001), which may contribute to age-induced loss of hearing.

### Purinergic Pathways

Adenosine is an endogenous nucleoside present in all cells of the body that exerts its pharmacologic effects by activation of purine adenosine receptors, also present in the HCs, supporting Deiters’ cells and SGNs (Vlajkovic et al., 2007, 2009). Adenosine is released from tissue in response to stress as a cytoprotectant, augments antioxidant defense, increases oxygen supply, improves blood flow, inhibits the release of neurotransmitters, stabilizes cells by stimulating K^+^ channels and inhibiting Ca^2+^ channels, triggers anti-inflammatory responses, and promotes anti-apoptotic pathways (Linden, 2005; Jacobson and Gao, 2006; Fredholm, 2007). It is administered as analgesics, vasodilator and anti-arrhythmia agents (compound summary is available through the PubChem Substance and compound database through the chemical structure identifier CID60961 (NCBI)),^1^ and has been shown to exert a protective effect to noise-induced oxidative cochlear damage (Hu et al., 1997; Hight et al., 2003; Fredholm, 2007). Local or systemic administration of selective A_1_ adenosine receptor agonists such as 2-chloro-*N*^6^-cyclopentyladenosine (CCPA) and adenosine amine congener (ADAC), has further shown promise in ameliorating noise- and cisplatin-induced cochlear injury (Vlajkovic et al., 2010, 2014). Most importantly, unlike most other therapeutic agents currently in trial which offer prophylactics, ADAC can be both otoprotective and therapeutic; systematic administration after the cessation of noise exposure improved hearing thresholds with increased survival of HCs and reduced expression of oxidative stress markers in the cochlea (Vlajkovic et al., 2010), with a therapeutic window of ≤24 h post exposure at doses >50 μg/kg and pharmacokinetic half-life of 5 min (Vlajkovic et al., 2014). Vlajkovic and colleagues also investigated if enhancing extracellular adenosine by selectively inhibiting adenosine kinase, the key enzyme in adenosine metabolism, may provide protection against ARHL in C57/B6 mice (Vlajkovic et al., 2011). The study found continuous inhibitor administration from early and late adulthood (3- and 6-month old) to reduce hearing loss at 9-months of age.

A theory much revisited recently is the role of intrinsic feedback pathways providing endogenous cochlear tissue protection against noise damage. Purinergic signaling through ATP activation of the ATP-gated ion channel P2X_2_ receptor subunit expressed in the sensory HCs and epithelial cells lining the scala media of the cochlea is known to modulate cochlear function through regulating ion homeostasis (Housley et al., 1999, 2002, 2009; Thorne et al., 2002; Wang et al., 2003c). In a recent study, Housley and colleagues have shown that ATP is released into the cochlear partition upon sound exposure, activating P2X_2_ receptors, which reduce the sensitivity of the HCs through K^+^ shunting (Housley et al., 2013). This purinergic regulation of hearing sensitivity was revealed by the absence of noise-induced temporary threshold shift (TTS) in P2X_2_ receptor knockout mice. P2X_2_ receptor knockout mice also showed higher threshold shifts in response to moderate noise exposure and more substantial permanent loss of hearing sensitivity compared to their wild-type littermates, supporting the otoprotective role of P2X_2_ receptor signaling pathway. Noise-induced ATP release and P2X_2_ is rapidly regulated by the ectonucleoside triphosphate diphosphohydrolases (E-NTPDase) family which hydrolyze extracellular nucleotides. NTPDase3 is expressed in the SGNs and the synaptic regions of the inner and outer HCs, which is upregulated in noise-exposed rat cochleae (Vlajkovic et al., 2006). Disrupted P2X_2_ signaling in humans and the total lack of P2X_2_ signaling in P2X_2_ knockout mice also showed exacerbated ARHL, evident in a flattened sensory epithelium in the basal organ of Corti (Yan et al., 2013).

## Mechanisms of Cochlear Neuronal Damage

Cochlear neurons are frequently lost secondary to HC loss. This is generally thought to be due to loss of neurotrophin exposure provided by the HCs, and can occur rather slowly, especially in humans. Primary loss of SGNs in the absence of HC damage also occurs. One cause of this is thought to be neurotropic viruses, but proof for this is difficult to obtain. However, an established mechanism of noise-induced cochlear neuronal damage is the excess release of the excitatory neurotransmitter glutamate at the inner HC afferent synapse. Glutamate excitotoxicity resulting from excessive glutamate release following noise overstimulation leads to an influx of cations such as Ca^2+^ across the post-synaptic membrane. Such created osmotic imbalance, resulting in cellular swelling (vacuolization) of the SGNs and the afferent dendritic endings that synapses onto the inner HCs (Pujol and Puel, 1999; Wang et al., 2002). This process has been replicated by administration of exogenous glutamate receptor agonists including AMPA and kainite to the cochlea (Ruel et al., 2000; Le Prell et al., 2004). Secondary to this cellular degeneration is excessive Ca^2+^ influx, leading to calcium-dependent caspase-mediated apoptosis by intrinsic (mitochondria-mediated) pathway (Puel et al., 1998; Pujol and Puel, 1999; Ruel et al., 2007).

Noise exposure can lead to loss of many afferent synapses and degeneration of type 1 SGNs within weeks and months after noise exposure (Kujawa and Liberman, 2009), even when the exposure results in no permanent threshold shift or HC loss. This form of damage is thought to result in degraded ability to process and analyze auditory input, and is almost certainly caused by glutamate toxicity (Pujol et al., 1990). Cellular mechanisms leading to SGN loss after noise are less clear, but Selivanova et al. (2007) found that JNK levels are increased and AKT levels decreased in SGNs after moderate noise exposure in guinea pigs. Interestingly, Zuccotti et al. (2012) found that conditional deletion of BDNF in HCs and SGNs lead to reduced activity and reduced numbers of afferent synapses on inner HCs, but also to reduced noise-induced loss of the remaining synapses. These data indicate that neurotrophic support is critical to both the function and survival of afferent cochlear synapses.

## Mechanisms of Central Auditory Damage

The mechanisms that contribute to central auditory deficits associated with noise damage and aging have received much less attention than those underlying cochlear damage. However, there is evidence for overlap of some mechanisms. For example, since glutamate is the major excitatory neurotransmitter of the central auditory pathway, glutamate toxicity following noise has been proposed to explain central auditory neuronal losses (Feng et al., 2012). Similarly, gradual accumulation of glutamate-related damage over time may contribute to age-related declines in central auditory neuronal populations (Tadros et al., 2007), perhaps exacerbated by declining inhibitory inputs (Caspary et al., 2008). Similarly, ROS accumulation due to noise-induced activity has been suggested to damage central neurons, while the accumulation of ROS damage with age may underlie damage to the central auditory pathway as it is thought to do in other parts of the central nervous system (Mattson and Magnus, 2006). It has also been suggested that deafferentation of central auditory neurons due to loss of HCs and SGNs reduces leads to transneuronal degeneration, due to loss of neurotrophic support (e.g., Feng et al., 2012).

Clues as to mechanisms of central auditory damage can also be inferred from research on the senescence-accelerated mouse model. ROS damage has also been implicated in age-related changes in the central nervous system of the senescence-accelerated mice (Wang et al., 2014). They noted brain increases in the oxidative stress-related marker malondialdehyde and decreases in the protective enzyme SOD. There is also evidence to implicate inflammation. Hasegawa-Ishii et al. (2015) observed enhanced expression of cytokines and chemokines that would be expected to increase inflammation and the recruitment of inflammatory cells in the brains of senescence-accelerated mice. Similar effects seem likely to operate in the central auditory system, and may also occur in the cochlea.

## Discussion

### Balance of Damage and Survival Signaling Determines Cochlear Damage

**Figure 2:**
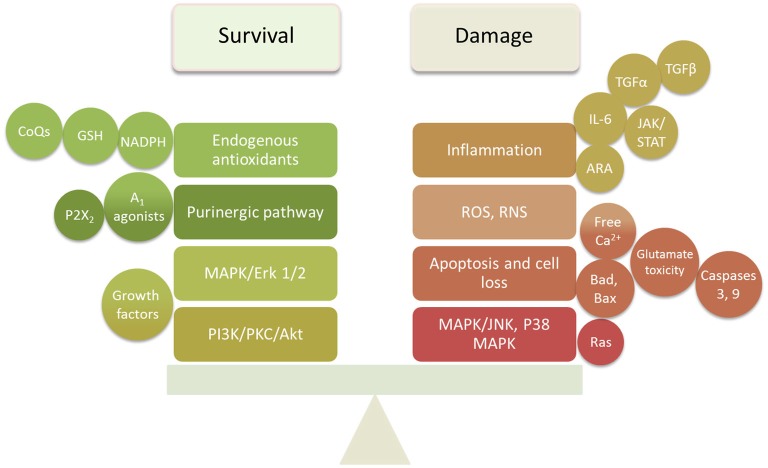
**Summary diagram of the survival and damaging factors in the cochlea that require balancing to prevent or delay SNHL**.

The studies reviewed above indicate that damage to cochlear tissues can involve a variety of mechanisms. Many of these are specific to the particular mode of damage and the cell types involved. However, there are common pathways that appear to operate across several different damage etiologies, including presbycusis. These include ROS accumulation, MAPK signaling, and apoptosis pathways. It is also clear that pathogenic stimuli activate not only damage mechanisms in cochlear cells, but also pro-survival processes that attempt to rescue cells. It is likely the balance of pro-death vs. pro-survival signaling that determines the fate of cells. This provides two potential mechanisms by which to protect cochlear tissues from damage: inhibition of damage-promoting processes and enhancement of survival-promoting mechanisms (Figure 2).

### ARHL and the Cumulative/Synergistic Damage Hypothesis

There is considerable disagreement regarding the mechanisms of ARHL. There is evidence to suggest that presbycusis includes the accumulation of cochlear damage from various sources, and especially that of noise exposure. A study of hearing in Mabaan natives of Sudan in 1962 (Rosen et al., 1962; Bergman, 1966), a pre-industrial people, revealed superior hearing in older individuals to that observed in US population studies. However, it can be argued that this difference might be caused by genetic differences between the populations. Goycoolea et al. (1986) studied the hearing of natives of Easter Island, 45 years of age or older, who at the time lived in a largely pre-industrial society. Included in the study were natives who had emigrated to mainland Chile and spent varying amounts of time in modern society. They found that hearing in males that had lived or were living in Chile was significantly worse than that of males who had lived their entire lives on Easter Island, and that this difference was related to years lived in modern society.

Another issue for presbycusis involves synergy between different etiologies of cochlear damage. The involvement of common pathways in some forms of cochlear pathogenesis suggests that the combination of two or more forms may lead to even greater damage. There is evidence that this can occur even when the exposures are not concurrent. Ryan and Bone (1982) found that prior exposure to noise that induced only TTS rendered animals more susceptible to later HC and hearing loss induced by aminoglycoside exposure. This finding also argues for the accumulation of damage from various etiologies as a contributing factor in presbycusis.

**Figure 3:**
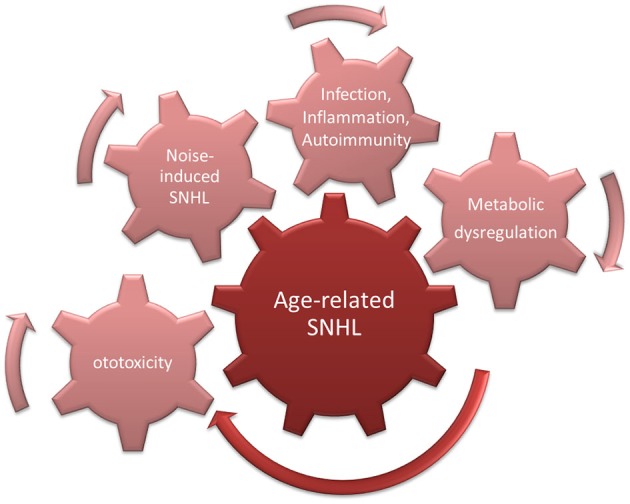
**Cumulative/synergistic damage hypothesis of age-related hearing loss (ARHL)**. Causes of SNHL that accumulate over a lifetime combine with endogenous susceptibility to exacerbate age-related SNHL.

However, even in studies of pre-industrial societies, it was found that hearing decreased even in individuals for whom noise exposure was presumably restricted by lifestyle (Rosen et al., 1962; Bergman, 1966; Goycoolea et al., 1986). This suggests that a “pure” form of presbycusis exists. This could be driven by increased ROS accumulation in cochlear cells, due for example to age-related decreases in mitochondrial membrane integrity (Shigenaga et al., 1994). This would be expected to lead to MAPK cell damage signaling and enhanced probability of apoptosis (Figure 3).

### Implications for Therapeutic Interventions

Because presbycusis is a predictable form of hearing loss and occurs over a long span of time, the therapeutic intervention for its prevention is feasible. As noted above, there are at least two potential strategies for therapeutic intervention to reduce cochlear damage. One is to inhibit processes or pathways that lead to the damage of cochlear cells. The other is to enhance processes that enhance cochlear cell survival. Both of these strategies have been attempted, with varying degrees of success.

Given the role of the MAPK-JNK signaling pathway in acoustic trauma and aminoglycoside induced HC apoptosis, studies have investigated the use of pharmacological blockers to protect sensorineural tissue from apoptosis (Pirvola et al., 2000; Hu et al., 2002a; Wang et al., 2003b). In particular, a peptide inhibitor of JNK kinase-mediated mitochondrial cell death pathway, D-JNKI-1 peptide, has shown promises in protecting the cochlea against noise-induced and aminoglycoside induced HC loss when delivered directly into the scala tympani or locally to the round window membrane (Wang et al., 2003b, 2007; Eshraghi et al., 2007). D-JNKI-1 has been developed under the name of AM-111 (Xigen/Auris Medical); a Phase 2b clinical trial has been completed in 2012 in three European countries and have shown promise for the treatment of acute SNHL (ClinicalTrial.gov Identifier: NCT00802425). Other experimental otoprotective compounds and potential gene therapy options against SNHL have been recently reviewed in Wong et al. (2013a).

Perhaps the most promising therapeutic interventions thus far are the various antioxidants investigated as ARHL prophylaxis to enhance cochlear defense against oxidative stress. Seidman et al. (2000) found that oral supplementation of acetyl-L-carnitine or α-lipoic acid delayed ARHL in the aging rat model. In a study that investigates the involvement of mitochondrial proapoptotic Bcl-2 family member *Bak* in ARHL, Someya et al. (2009) looked at the effects of long-term dietary supplementation of 17 antioxidant compounds on ARHL development in C57BL/6J mice. They found compounds that act as mitochondrial antioxidants to delay high-frequency threshold elevation and maintain sensorineural tissue survival. Of which α-lipoic acid and N-acetyl-L-cysteine (NAC) are thiol compounds that are shown to reduce mitochondrial ROS production, and coenzyme Q10 is an essential mitochondrial electron transfer chain component that acts as a mitochondrial antioxidant. An antioxidant cocktail of L-cysteine-glutathione mixed disulfide (L-CySSG), ribose-cysteine, NW-nitro-L-arginine methyl ester (L-NAME), vitamin B12, folate, and ascorbic acid as a dietary supplement early in life has been shown to reduce age-related threshold shifts in C57BL/6 mice (Heman-Ackah et al., 2010). However, in CBA/J mice which do not have the *Cdh23^ahl^* allele, an antioxidant-rich diet fails to provide advantage (Sha et al., 2012). In humans, a recent study correlated the dietary intake of β-carotene, vitamin C, vitamin E, and magnesium to a hearing threshold of ~2500 US adults (Choi et al., 2014), has found higher intakes of the antioxidants and magnesium to associate with a lower hearing threshold, interpreted as less hearing loss. For the time being, an antioxidant rich diet with physical protection against overexposure of noise seems a sensible and feasible prevention to ARHL prone individuals.

## Conclusions

With irreversible damages of the sensorineural tissue in the cochlea accumulating over a lifetime, ameliorating age-related SNHL seems daunting. However, we now understand far more of the cellular mechanisms leading to sensorineural damage, and the survival cellular signaling elements that can counterbalance and prevent it. Therapeutics that provide protection in the form of oral otoprotective formulations with antioxidant or other actions, or long-term local application of compounds that inhibit cell damage pathways or promote cell survival can readily be envisioned. Although a single mechanism responsible for ARHL is yet to be identified, and is rather unlikely with the multifactorial and cumulative nature of the damage, it is certain that ongoing research efforts to advance our understanding of SNHL, for example with transgenic animal models, high-throughput genome sequencing and proteomics, will further support evidence-based treatment. Advances in drug delivery to the inner ear will further support therapies for prebycusis.

## Conflict of Interest Statement

The authors declare that the research was conducted in the absence of any commercial or financial relationships that could be construed as a potential conflict of interest.

## Footnotes

^1^NCBI National Center for Biotechnology Information; PubChem Compound Database; CID=60961. In: PubChem Compound Database.
